# 

**Published:** 1887-02

**Authors:** 


DISEASES OF THE BLOOD AND NUTRITION, AND INFECTIOUS DISEASES; being Vol. IV. of "A Handbook of Practical Medicine," by Dr. Hermann Eichhorst, and Vol. XII. of Wood's Library for 1886 (completing the set, price of set, $15.00). Illustrated. New York, William Wood & Company.

These two works complete the series of the "Library" for 1886. We have not given them a critical examination, but a casual glance reveals the fact that in making the selection the publishers exercised commendable discretion and taste in their endeavor to give great value for little money, to give variety, and at the same time to have all of the works of a high order of excellence.

